# A randomized controlled Phase I de-escalation trial of molnupiravir and nirmatrelvir/ritonavir combination for mild-moderate SARS-CoV-2 infection

**DOI:** 10.1093/jac/dkag180

**Published:** 2026-06-16

**Authors:** Saye H Khoo, Richard FitzGerald, Christopher J Edwards, Shazaad Ahmad, Geoffrey Saunders, Laura J Else, Victoria Shaw, Pavel Mozgunov, Joshua Northey, Laura Dickinson, Emma Knox, Amanda Buadi, Colin Hale, Helen E Reynolds, Calley Middleton, Katie Bullock, Lauren Walker, Michelle Tetlow, Rebecca Lyon, Jennifer Gibney, Alieu Amara, William Greenhalf, Abigail Burdon, Jan Dixon, Thomas Jaki, Justin Chiong, David G Lalloo, Andew Owen, Michael Jacobs, Thomas Fletcher, Gareth Griffiths, the AGILE CST-8 study group, Nicholas Paton, Fred Hayden, Janet Darbyshire, Amy Lucas, Ulrika Lorch, Andrew Freedman, Richard Knight, Steven Julious, Thomas Edwards, Christopher Myerscough, Nuala Tainton, Oliver Edwards, Kerensa Thorne, Sujan Dily Penchala, Rachel Carter, Callum Kelly, Kate Dodd, Callum Docherty, Charlotte Boe, Alasdair Munro, Dan Owens, Mihaela Pacurar, Gavin Babbage, Liza Shiner-Clark

**Affiliations:** Centre for Experimental Therapeutics (TherEx), University of Liverpool, Liverpool, UK; NIHR Liverpool Clinical Research Facility, NHS University Hospitals of Liverpool Group, Liverpool, UK; Centre for Experimental Therapeutics (TherEx), University of Liverpool, Liverpool, UK; NIHR Liverpool Clinical Research Facility, NHS University Hospitals of Liverpool Group, Liverpool, UK; NIHR Southampton Clinical Research Facility, University Hospital Southampton NHS Foundation Trust, Southampton, UK; NIHR Manchester Clinical Research Facility, University of Manchester, Manchester, UK; Southampton Clinical Trials Unit, University of Southampton and University Hospital Southampton NHS Foundation Trust, Southampton, UK; Centre for Experimental Therapeutics (TherEx), University of Liverpool, Liverpool, UK; Clinical Directorate, University of Liverpool, Liverpool, UK; MRC Biostatistics Unit, University of Cambridge, Cambridge, UK; Southampton Clinical Trials Unit, University of Southampton and University Hospital Southampton NHS Foundation Trust, Southampton, UK; Centre for Experimental Therapeutics (TherEx), University of Liverpool, Liverpool, UK; Southampton Clinical Trials Unit, University of Southampton and University Hospital Southampton NHS Foundation Trust, Southampton, UK; NIHR Southampton Clinical Research Facility, University Hospital Southampton NHS Foundation Trust, Southampton, UK; NIHR Liverpool Clinical Research Facility, NHS University Hospitals of Liverpool Group, Liverpool, UK; Centre for Experimental Therapeutics (TherEx), University of Liverpool, Liverpool, UK; Southampton Clinical Trials Unit, University of Southampton and University Hospital Southampton NHS Foundation Trust, Southampton, UK; Molecular & Clinical Cancer Medicine, University of Liverpool, Liverpool, UK; Centre for Experimental Therapeutics (TherEx), University of Liverpool, Liverpool, UK; NIHR Liverpool Clinical Research Facility, NHS University Hospitals of Liverpool Group, Liverpool, UK; Centre for Experimental Therapeutics (TherEx), University of Liverpool, Liverpool, UK; NIHR Liverpool Clinical Research Facility, NHS University Hospitals of Liverpool Group, Liverpool, UK; NIHR Liverpool Clinical Research Facility, NHS University Hospitals of Liverpool Group, Liverpool, UK; Centre for Experimental Therapeutics (TherEx), University of Liverpool, Liverpool, UK; Molecular & Clinical Cancer Medicine, University of Liverpool, Liverpool, UK; MRC Biostatistics Unit, University of Cambridge, Cambridge, UK; Centre for Experimental Therapeutics (TherEx), University of Liverpool, Liverpool, UK; MRC Biostatistics Unit, University of Cambridge, Cambridge, UK; Computational Statistics, University of Regensburg, Regensburg, Germany; Centre for Experimental Therapeutics (TherEx), University of Liverpool, Liverpool, UK; Clinical Sciences, Liverpool School of Tropical Medicine, Liverpool, UK; Centre of Excellence for Long-Acting Therapeutics, University of Liverpool, Liverpool, UK; Clinical Sciences, Liverpool School of Tropical Medicine, Liverpool, UK; Keble College, University of Oxford, Oxford, UK; Clinical Sciences, Liverpool School of Tropical Medicine, Liverpool, UK; Southampton Clinical Trials Unit, University of Southampton and University Hospital Southampton NHS Foundation Trust, Southampton, UK

## Abstract

**Objectives:**

The AGILE CST-8 (NCT04746183) Phase I de-escalation trial evaluated the safety and tolerability of combination molnupiravir and nirmatrelvir/ritonavir for mild-moderate COVID-19.

**Methods:**

Adult outpatients with SARS-CoV-2 infection within 5 days of symptoms were randomly assigned 2:1 to receive molnupiravir [starting at 800 mg twice daily (BD) reducing to 600 and 400 mg if necessary] in combination with nirmatrelvir (300 mg)/ritonavir (100 mg) BD for 5 days versus standard of care. Using a dose de-escalation, open-label, Bayesian adaptive Phase I trial, a combination dose was considered unsafe if the probability of 30% or greater dose-limiting toxicity risk (DLT—the primary outcome) over standard of care was 25% or higher. Secondary endpoints included tolerability, clinical progression, pharmacokinetics and virological responses.

**Results:**

Of 49 participants screened, 24 were enrolled (16 combination, 8 standard of care) between January 2023 and September 2023. For the primary endpoint, to Day 11, no participant starting molnupiravir at 800 mg BD in combination with nirmatrelvir/ritonavir reported a DLT by Day 11 (primary endpoint) or by Day 29; dose de-escalation was not required. No participants reported severe adverse events (grade ≥3). Although proportions of swab PCR negativity at Day 5 and Day 11 were not statistically different, faster initial viral clearance was observed with treatment. Penetration of nirmatrelvir into saliva, nasal secretions and tears was 19%, 65% and 91% that of plasma.

**Conclusions:**

Molnupiravir in combination with nirmatrelvir/ritonavir was safe and well-tolerated; later phase trials should evaluate combination therapy at currently recommended doses for each drug.

## Introduction

In profoundly immunosuppressed patients, chronic persisting or relapsing severe acute respiratory syndrome coronavirus 2 (SARS-CoV-2) infection despite currently approved antiviral treatments^1^ may create a window of opportunity for the evolution of new variants, and the emergence of drug-resistant mutations. Indeed, such a scenario has been described for remdesivir and nirmatrelvir,^2–6^ although the prevalence of drug resistance is not currently widespread.^7^ The failure of current antiviral therapy to eradicate chronic infection in these patients, coupled with evidence for a more prolonged persistence of SARS-CoV-2 in blood^8^ and gut tissues, suggests that greater antiviral potency and a higher genetic barrier to resistance is required. Combinations with two^9^ or three^10^ antivirals have been empirically administered for persistent SARS-CoV-2 infections, but combination therapy has not been systematically evaluated in randomized trials.

Combination therapy with molnupiravir and nirmatrelvir/ritonavir is associated with greater *in vitro* potency^11^ and greater reductions in viral shedding and replication and reduced lung pathology in animal models.^12,13^ However, such an approach is not without risk: any increase in toxicity cannot be adequately characterized in such complex patients, the efficacy of such combinations needs to be properly tested, and the correct use of each drug at full dose within any combination cannot be automatically assumed. Further, in the case of combinations involving molnupiravir (which induces hypermutation) there is potential risk of inducing mutations that become enriched for drug resistance under selective pressure from a second antiviral. In macaques treated with molnupiravir, mutations in 3CL protease (including T21L, L50F, A173V and P252L; conferring resistance to nirmatrelvir) were observed but combination molnupiravir plus nirmatrelvir was not associated with selective enrichment of these mutations.^13,14^

AGILE is the UK early-phase trials platform evaluating experimental COVID-19 antivirals^15^ through a master protocol and individual candidate-specific trials. AGILE undertakes dose optimization using a Bayesian model to characterize the relationship between dose and toxicity. In this study (CST-8), we undertook an open-label, randomized, Phase I dose de-escalation trial to assess the safety and tolerability of the drug combination of molnupiravir and nirmatrelvir/ritonavir in patients with mild-moderate SARS-CoV-2 infection. The dose de-escalation protocol was applied to molnupiravir only, since dose reduction of nirmatrelvir would have significantly altered the ratio of pharmacokinetic boosting with ritonavir, and *in vitro* evidence for synergy^16^ of this combination may reduce the risk of under-dosing with molnupiravir.

## Methods

### Trial design and participants

AGILE CST8 (NCT04746183) was an open-label, randomized, controlled, de-escalation Bayesian adaptive Phase I trial in adults with COVID-19 with the primary objective to determine the safety and tolerability and recommended Phase II dose of molnupiravir in combination with nirmatrelvir/ritonavir. Eligible participants were aged ≥18 years with lateral flow–positive SARS-CoV-2 infection, were within 5 days of symptom onset, free of uncontrolled chronic conditions, ambulant in the community with mild or moderate disease, and regardless of vaccination status. Women of childbearing potential and men with female partners of childbearing potential were required to use a highly effective method of contraception for the duration of the treatment and for 6 weeks following the last dose. Exclusion criteria included pregnant or breastfeeding women, swallowing difficulties, known medical history of liver disease, receiving dialysis or known moderate to severe renal impairment (chronic kidney disease stage 4 or 5), oxygen saturation <92% on room air (or their standard home oxygen supplementation), ALT >5 times upper limit of normal, known allergy to any study medication, or having received any other experimental agents within 30 days of first dose of study drug (use of contraindicated co-medications as defined in the Summary of Product Characteristics for molnupiravir^17^ and nirmatrelvir/ritonavir^18^ was not permitted; other medications were permitted at physician’s discretion guided by recommendations from the Liverpool COVID-19 Drug Interactions tool^19^).

### Ethics

The study protocol was reviewed and approved by the UK Medicines and Healthcare Product Regulatory Agency (EudraCT 2020–001860-27) and West Midlands Edgbaston Research Ethics Committee (20/WM/0136). All participants provided written informed consent.

### Randomization, blinding and allocation

Participants were allocated 2:1 (block randomization with no stratification) to either molnupiravir plus nirmatrelvir/ritonavir or standard of care, with no masking of allocation except for laboratory testing for virological and pharmacokinetic evaluation. Participants received molnupiravir (Lagevrio^®^) at its licensed dose of 800 mg in combination with nirmatrelvir 300 mg/ritonavir 100 mg (Paxlovid^®^) twice daily (12 h apart ± 4 h, without regard to food) for 5 days as full doses at the start, with a de-escalation protocol reducing molnupiravir in decrements to 600 mg twice daily (BD), then 400 mg BD if required. Nirmatrelvir/ritonavir doses were fixed throughout the trial owing to the limited options for de-escalation. Participants were allowed flexibility in dosing by 4 h; missed doses beyond this time were omitted. Standard of care included symptomatic relief such as antipyretics. No participant in the standard of care arm received any antiviral therapy. All participants attended for review on Days 3, 5 and 11, bringing study medication bottles for drug accountability.

### Procedures

Up to 24 participants were block randomized (2:1 with no stratification) to combination therapy for 5 days or standard of care, with recruitment in four cohorts of 6 participants each (4 combination, 2 standard of care) and review of safety and tolerability between cohorts. Participants were seen in clinic at baseline (Day 1) and Days 3, 5, 11 and 29 with telephone follow-up (and home swab collection) on Days 2 and 4. A two-parameter Bayesian dose de-escalation model was utilized^20^ (Supplementary Material S1, available as Supplementary data at *JAC* Online) to recommend the next dose level that targeted a safe dose with an additional risk of dose-limiting toxicities (DLTs) of 20% (the target interval of 15%–25%) above standard of care. A dose was deemed unsafe if there was a ≥25% probability that the risk of toxicity was 30% higher than standard of care. As an additional stopping rule if the highest dose combination (800 mg molnupiravir with nirmatrelvir/ritonavir) had a probability of greater than 47.5% of being unsafe (i.e. >47.5% probability that the risk of toxicity was 30% higher than standard of care), then the study would halt due to safety concerns. A Safety Review Committee reviewed dose-limiting toxicities (up to at least Day 11 of follow-up) at the end of each of the first two cohorts (and if required, cohort 3) in order to recommend de-escalation of the molnupiravir dose or else proceeding to full recruitment if the probability of the combination being safe was considered high as guided by safety data and the Bayesian dose-toxicity model.

### Outcomes

The primary outcome was DLTs, defined as any adverse event Common Terminology Criteria for Adverse Events (CTCAE) version 5 grades 3 and above, up to and including Day 11. Secondary outcomes for safety included adverse events (AEs), serious adverse events (SAEs), physical findings, vital signs (heart rate, blood pressure, respiratory rate, temperature and oxygen saturation), time to negative PCR and viral load reduction [estimated using the log_10_ mean pseudo-concentration of the three genes (N, ORF and S, if amplified)^21^], laboratory parameters including blood and urinary (with plasma, saliva, tears and nasal swabs for pharmacokinetics at Days 1 and 5), ECG, and the number of deaths and hospitalizations up to Day 29.

### Virological characterization

Serial swabs (sampled from the oropharynx, then mid-turbinate space) were collected in DNA/RNA Shield (Zymo Research #R1100, USA) as previously described^21^ at baseline (Day 1), then Days 2, 3, 4, 5 and 11 (with self-collection at Days 2 and 4). Following extraction, viral RNA was quantified using the TaqPath COVID-19 RT-PCR Kit (ThermoFisher Scientific, Waltham, MA, USA), with thresholds for each amplicon (S-gene, N-gene and ORF1) adjusted to give a threshold cycle of 32 with a control of 25 templates per reaction. Time of negativity within an amplicon was determined by the time of the first of two consecutive readings below the limit of detection (cycle threshold of 32 or more) where at least two amplicons were concordant. If all three amplicons differed, the median time to negative PCR was used. If only two amplicons were evaluable (e.g. if the third was censored), the later time of the two was used. Where only one amplicon was evaluable, time to negative PCR was censored at the last PCR measurement. In the event of S-gene amplification failure, the S-gene was considered censored at Day 11 and the rules above applied. Viral titre was quantified by estimating a viral ‘pseudoconcentration’ (expressed as log_10_ copies of template per reaction) as previously described.^21^

### Pharmacokinetic evaluation

Plasma was collected on Day 1 and Day 5 with sampling pre-dose (0 h), 0.5, 1, 2 and 4 h post-dose for quantification of β-D-*N*_4_-hydroxycytidine (NHC; the active metabolite of molnupiravir), nirmatrelvir and ritonavir as previously described.^22,23^ Briefly, plasma extracts [obtained after protein precipitation using 3:1 (v/v) acetonitrile to plasma] were chromatographically separated using a reverse-phase Atlantis dC_18_ column (Waters, UK) followed by LC-MS (AB Sciex 4500, Framingham, USA) analysis. The lower limits of quantification were as follows: NHC 2.5 ng/mL, nirmatrelvir 2.5 ng/mL and ritonavir 1.25 ng/mL. Non-compartmental analyses of truncated profiles with AUC calculated using the linear trapezoidal rule were compared using geometric means (95% CI) (Phoenix WinNonlin v.8.3, Certara, USA).

Drug penetration into upper airway fluids may be important for local sterilization and inform strategies for prevention. We also measured concentrations of nirmatrelvir and ritonavir in saliva, nasal mucosa and tears at steady-state on Day 5; samples were collected (with paired plasma) pre-dose, 30 min, 1 h, 2 h and 4 h post-dose.^24^ Saliva was centrifuged from Salivette^TM^ swabs (Sarstedt Ltd, UK), chewed for 60 s. Nasal secretions were collected using Synthetic Absorptive Matrix (SAM) strips (Mucosal Diagnostics, UK) applied against the surface of the inferior turbinate of each nostril for 60 s—weights (to the nearest 0.1 mg) were recorded before and after sampling. Tears were collected using Schirmer Tear Test strips inserted in each lower eyelid for 5 min with approximate volumes (to the nearest µL) recorded using the graduated markings on the strip (1–35 µL). Drug extraction, and measurement using tandem LC-MS/MS, have been previously described.^24^ The lower limits of quantitation for nirmatrelvir (saliva, tears, nasal secretions) were 2.5 ng/mL, 0.04 ng/sample and 0.04 ng/sample, respectively, and for ritonavir in saliva it was 1.25 ng/mL (ritonavir was not measured in tears or nasal secretions due to the very low levels seen in saliva, and because it is not an active component of the regimen for efficacy).

### Safety assessments

Safety was evaluated at specific time points throughout the trial using CTCAE version 5 with real-time SAE reporting. Study visits at baseline and Days 1, 3, 5 and 8 included collection of safety bloods (including full blood count, biochemistry, liver and renal function), clinical assessment and measurement of vital signs. At these visits, and additionally at Days 2, 4 and 29, symptom, health and medication questionnaires were administered.

### Statistics

We used an adaptive model-based dose-finding design,^20^ with all analyses conducted on an intention-to-treat basis except for the safety analysis, which included participants who received at least one dose of allocated treatment. There was no imputation of missing data, data transformations (apart from log_10_ viral load) or adjustment for multiplicity. The primary analysis of DLTs up to and including Day 11 used a two-parameter Bayesian dose de-escalation model with a prior estimate of the DLT risk for a participant on standard of care assumed to be 10%, with results presented as the number of DLTs and posterior point estimates and 95% equal-tail credible intervals of the risk of a DLT for each dose (including standard of care) (Supplementary Material S2). As a secondary analysis the model was applied to all data available, i.e. DLTs up to Day 29. Descriptive analyses of baseline characteristics and other endpoints were summarized using means, medians and proportions with corresponding IQRs or 95% CIs as appropriate. All analyses were reported according to CONSORT 2010 and the International Council for Harmonisaton of Technical Requirements for Pharmaceuticals for Human Use E9 guidelines on Statistical Principles in Clinical Trials. All analyses were carried out in SAS v9.4 and Stata v16 except the Bayesian analyses, which were performed using packages available in R v4.0.2.

Viral load reductions from baseline were fitted to a Bayesian bi-exponential model^25^ to represent ‘fast’ decay at an initial stage of viral elimination and ‘persistent’ decay at a second stage of viral elimination. The mixed model is used to account for variability in slopes between participants. In the primary pre-specified analysis model it was assumed that treatment only affects the fast decay (a subsequent sensitivity analysis also evaluated for effects on both phases of viral clearance). With *t* defined as time (days), the primary bi-exponential model is given by


$$f_{vl}^{i}(t)=\log\;(\exp\;\{A_{0}+\theta_{i,1}-\gamma_{1}^{Z_{i}}\alpha e^{\theta_{i,2}}t\}+\exp\;\{B_{0}+\theta_{i,3}-\beta e^{\theta_{i,4}}t\})$$

where *A*_0_ and α are the population intercept and slope, respectively, for the fast decay stage and *B*_0_ and β are the population intercept and slope, respectively, for the persistent decay stage. The parameter γ_1_ represents the treatment effect coefficient, and *Z*_i_ is the treatment indicator taking the value of 1 if patient I receives the experimental treatment and 0 otherwise. The vector θ_i_ = (θ_i_,1, θ_i_,2, θ_i_,3, θ_i_,4)T is a random effect specific to patient i. Full details of the model including the pre-specified prior distribution are given in Supplementary Material S2. The posterior probability of the treatment effect coefficient being above 1 is used to declare whether there is a treatment effect on the viral slope. Based on the simulations, if this posterior probability is above 91% then the treatment effect can be declared controlling the rate of false-positive conclusion at 10%.

## Results

### Trial population

**Figure 1.**
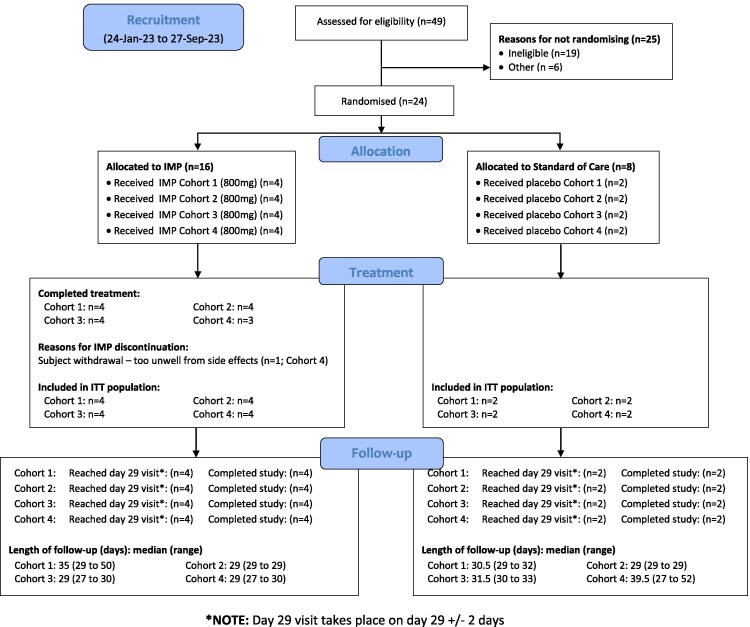
CONSORT diagram. IMP, investigational medicinal product; ITT, intention to treat.

Between January and September 2023, 49 potential participants (Figure 1) were screened; 25 were excluded; 8 could not comply with clinic visits, 6 could not be re-contacted, 5 did not fulfil symptom criteria, 5 had negative lateral flow tests, and 1 could not meet the contraception requirements. The remaining 24 eligible participants were randomly assigned (2:1 to receive combination antivirals or standard of care) across four sequential cohorts of 6 participants each (giving a total of 16 randomized to combination antivirals and 8 to standard of care), all of whom were included in the analysis.

The baseline characteristics of participants were broadly similar with regard to age, sex, vaccination status and BMI (Table 1). Median interval from symptom onset was 4 days in the combination arm, versus 3 days in the standard-of-care arm. A total of 94% (15/16) of combination participants completed the full treatment, with one starting treatment but withdrawing due to side effects (chills, fatigue, nausea and dysgeusia after receiving 4 doses out of 10—i.e. after 2 days of full treatment). All 24 participants had their Day 29 follow-up visit (±2 days).

**Table 1.** Demographic information at baseline: ITT population

Treatment arm:	IMP (800 mg) (*n* = 4)	IMP (800 mg) (*n* = 4)	IMP (800 mg) (*n* = 4)	IMP (800 mg) (*n* = 4)	Total IMP (800 mg) (*n* = 16)	Standard of care (*n* = 8)
Cohort:	Cohort 1	Cohort 2	Cohort 3	Cohort 4	All cohorts	All cohorts
Age at consent, y						
Median	34.5	65.5	39.0	27.0	35.5	35.0
Range	22.0 to 55.0	61.0 to 70.0	32.0 to 48.0	20.0 to 36.0	20.0 to 70.0	20.0 to 61.0
Sex, *n* (%)						
Male	1 (25.0)	1 (25.0)	4 (100)	0 (0.0)	6 (37.5)	1 (12.5)
Female	3 (75.0)	3 (750)	0 (0.0)	4 (100)	10 (62.5)	7 (87.5)
Ethnicity, *n* (%)						
White: English/Welsh/Scottish/Northern Irish/British	2 (50.0)	4 (100)	3 (75.0)	3 (75.0)	12 (75.0)	8 (100)
Any other White background	1 (25.0)	0 (0.0)	1 (25.0)	1 (25.0)	3 (18.8)	0 (0.0)
Any other Mixed/Multiple ethnic background	1 (25.0)	0 (0.0)	0 (0.0)	0 (0.0)	1 (6.3)	0 (0.0)
BMI, kg/m^2^						
Median	24.1	34.1	26.8	22.3	26.0	27.8
Range	21.9 to 31.7	24.2 to 40.2	22.8 to 27.4	20.2 to 28.0	20.2 to 40.2	21.2 to 39.7
Number of days experiencing COVID-19 symptoms prior to randomization, *n* (%)^a^						
1	0 (0.0)	0 (0.0)	0 (0.0)	0 (0.0)	0 (0.0)	1 (12.5)
2	0 (0.0)	0 (0.0)	0 (0.0)	0 (0.0)	0 (0.0)	1 (12.5)
3	0 (0.0)	2 (50.0)	2 (50.0)	2 (50.0)	6 (37.5)	3 (37.5)
4	2 (50.0)	2 (50.0)	2 (50.0)	1 (25.0)	7 (43.8)	1 (12.5)
5	2 (50.0)	0 (0.0)	0 (0.0)	1 (25.0)	3 (18.8)	2 (25.0)

IMP, investigational medicinal product; ITT, intention-to-treat.

Percentages are based on the number of participants in the study arm.

^a^Calculated as randomization date − symptom onset date.

### Primary analysis

**Figure 2.**
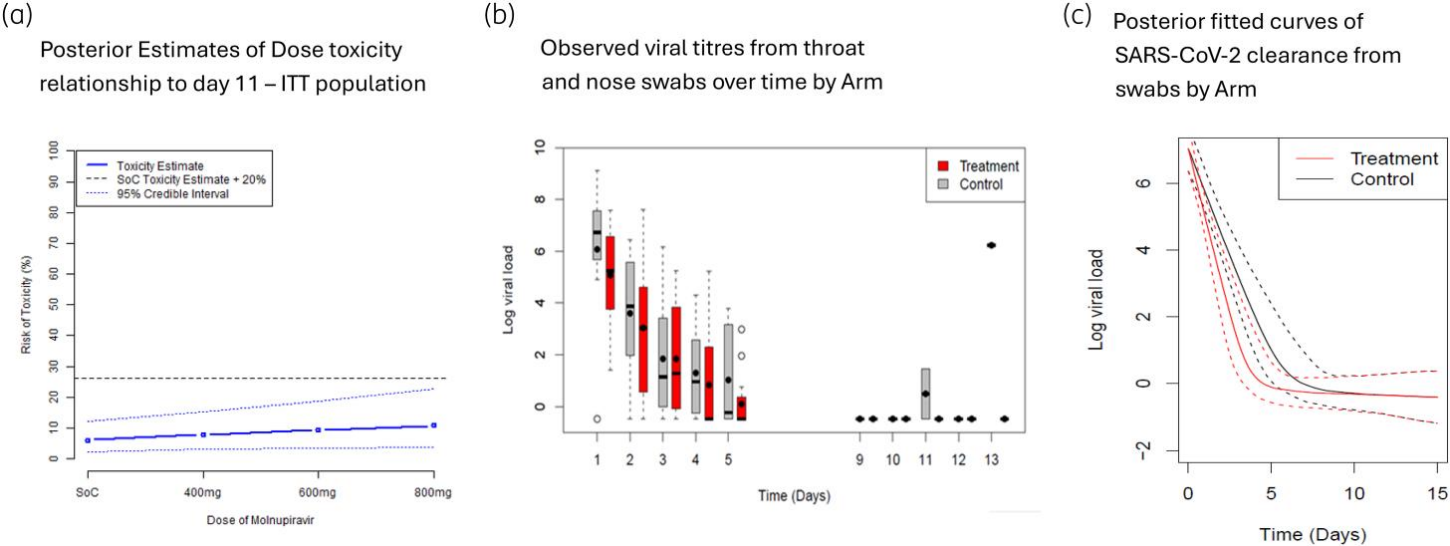
Bayesian estimates of dose-toxicity (primary endpoint) and virological efficacy Posterior estimates from participants in the combination (*N* = 16) and SoC (*N* = 8) arms. (a) Dose-Toxicity relationship (95% credible interval) to Day 11 (by intention-to-treat). (b) Viral titre (expressed as log10 ‘pseudocopies’/swab) derived from cycle threshold (CT) for three genes: N, S and ORF-1 as described in Methods. The mean CT of all three genes were used, with genes excluded if they did not amplify. Box plot shows viral load measurements by day, and by treatment group. Solid points give means and lines show medians. The box represents the 25th (Q1) and 75th (Q3) percentiles, and whiskers represent Q1 -1.5IQR and Q3+1.5IQR. (c) Biexponential model of virological clearance comparing slope parameter estimates of combination antiviral therapy versus Standard-of-care. The posterior time curves including all participants are shown. The median curves for treatment and control lines are given by solid lines and the dashed lines represent the 95% credible intervals. The treatment coefficient (γ1) between both arms is 1.57 (95% confidence 1.22 – 2.07) for treatment effect. See supplementary data S2 and text.

No participant in any cohort experienced a DLT (a grade 3 or above AE) (Table 2); consequently the Safety Review Committee confirmed all participants randomized to combination therapy should receive both drugs at full doses for 5 days. Data were analysed separately as well as pooled across cohorts to evaluate differences by arm. Bayesian model DLT point estimates, 95% credible interval and the target toxicity level of 20% over standard of care are shown in Figure 2. After the four cohorts for data up to Day 11, the 800 mg molnupiravir dose in the combination arm had an estimated DLT rate of 10.5% (equal-tail 95% credible interval of 3.8%–22.2%), with an estimated 4.6% additional toxicity over standard of care and a probability of additional toxicity of 30% over standard of care of 0%. Inclusion of DLTs recorded up to Day 29 (Supplementary data S2) into the model also supported a recommended Phase II dose of molnupiravir 800 mg with nirmatrelvir (300 mg)/ritonavir (100 mg) BD for 5 days.

**Table 2.** Overall toxicity summary by CTCAE version 5 term—safety population—all CTCAE version 5 grades 1 and 2^a^

Treatment arm:	IMP (800 mg) (*n* = 4)	IMP (800 mg) (*n* = 4)	IMP (800 mg) (*n* = 4)	IMP (800 mg (*n* = 4)	Total IMP (800 mg (*n* = 16)	Standard of care (*n* = 8)
Cohort:	Cohort 1	Cohort 2	Cohort 3	Cohort 4	All cohorts	All cohorts
Number of participants who experienced at least one AE, *n* (%)	4 (100%)	4 (100%)	3 (75%)	3 (75%)	14 (87.5%)	5 (62.5%)
Summary of AEs, *n* (%)						
Blood and lymphatic system disorders	0 (0.0)	1 (25.0)	0 (0.0)	0 (0.0)	1 (6.3)	0 (0.0)
B12 deficiency	0 (0.0)	1 (25.0)	0 (0.0)	0 (0.0)	1 (6.3)	0 (0.0)
Folate deficiency	0 (0.0)	1 (25.0)	0 (0.0)	0 (0.0)	1 (6.3)	0 (0.0)
Cardiac disorders	1 (25.0)	0 (0.0)	0 (0.0)	0 (0.0)	1 (6.3)	0 (0.0)
Palpitations	1 (25.0)	0 (0.0)	0 (0.0)	0 (0.0)	1 (6.3)	0 (0.0)
Ear and labyrinth disorders	0 (0.0)	2 (50.0)	0 (0.0)	0 (0.0)	2 (12.5)	0 (0.0)
Bilateral hearing impairment	0 (0.0)	1 (25.0)	0 (0.0)	0 (0.0)	1 (6.3)	0 (0.0)
Ear pain	0 (0.0)	1 (25.0)	0 (0.0)	0 (0.0)	1 (6.3)	0 (0.0)
Tinnitus	0 (0.0)	1 (25.0)	0 (0.0)	0 (0.0)	1 (6.3)	0 (0.0)
Vertigo	0 (0.0)	1 (25.0)	0 (0.0)	0 (0.0)	1 (6.3)	0 (0.0)
Gastrointestinal disorders	2 (50.0)	1 (25.0)	2 (50.0)	2 (50.0)	7 (43.8)	1 (12.5)
Diarrhoea	0 (0.0)	1 (25.0)	0 (0.0)	0 (0.0)	1 (6.3)	0 (0.0)
Dry mouth	0 (0.0)	0 (0.0)	1 (25.0)	0 (0.0)	1 (6.3)	0 (0.0)
Dyspepsia	0 (0.0)	0 (0.0)	1 (25.0)	0 (0.0)	1 (6.3)	0 (0.0)
Gastrointestinal discomfort	0 (0.0)	0 (0.0)	0 (0.0)	1 (25.0)	1 (6.3)	0 (0.0)
Nausea	1 (25.0)	0 (0.0)	0 (0.0)	1 (25.0)^a^	2 (12.5)	1 (12.5)^a^
Superficial ulcer/bruise in mouth	1 (25.0)	0 (0.0)	0 (0.0)	0 (0.0)	1 (6.3)	0 (0.0)
General disorders and administration site conditions	0 (0.0)	1 (25.0)	1 (25.0)	1 (25.0)	3 (18.8)	0 (0.0)
Chills	0 (0.0)	0 (0.0)	0 (0.0)	1 (25.0)^a^	1 (6.3)	0 (0.0)
Fatigue	0 (0.0)	0 (0.0)	1 (25.0)^a^	1 (25.0)^a^	2 (12.5)	0 (0.0)
Malaise	0 (0.0)	1 (25.0)	0 (0.0)	0 (0.0)	1 (6.3)	0 (0.0)
Infections and infestations	0 (0.0)	1 (25.0)	1 (25.0)	0 (0.0)	2 (12.5)	1 (12.5)
Folliculitis	0 (0.0)	0 (0.0)	1 (25.0)	0 (0.0)	1 (6.3)	0 (0.0)
Otitis media	0 (0.0)	1 (25.0)	0 (0.0)	0 (0.0)	1 (6.3)	0 (0.0)
Skin infection (impetigo)	0 (0.0)	0 (0.0)	0 (0.0)	0 (0.0)	0 (0.0)	1 (12.5)
Investigations	0 (0.0)	0 (0.0)	0 (0.0)	0 (0.0)	0 (0.0)	1 (12.5)
ALT increased	0 (0.0)	0 (0.0)	0 (0.0)	0 (0.0)	0 (0.0)	1 (12.5)
GGT increased	0 (0.0)	0 (0.0)	0 (0.0)	0 (0.0)	0 (0.0)	1 (12.5)
Musculoskeletal and connective tissue disorders	0 (0.0)	2 (50.0)	1 (25.0)	0 (0.0)	3 (18.8)	0 (0.0)
Back pain	0 (0.0)	1 (25.0)	1 (25.0)	0 (0.0)	2 (12.5)	0 (0.0)
Left shoulder pain	0 (0.0)	1 (25.0)	0 (0.0)	0 (0.0)	1 (6.3)	0 (0.0)
Muscle ache/weakness	0 (0.0)	0 (0.0)	1 (25.0)	0 (0.0)	1 (6.3)	0 (0.0)
Nervous system disorders	4 (100)	3 (75.0)	3 (75.0)	3 (75.0)	13 (81.3)	0 (0.0)
Brain fog/difficulty concentrating	0 (0.0)	0 (0.0)	1 (25.0)	0 (0.0)	1 (6.3)	0 (0.0)
Dizziness	1 (25.0)	0 (0.0)	0 (0.0)	0 (0.0)	1 (6.3)	0 (0.0)
Dysgeusia	4 (100)	3 (75.0)	3 (75.0)	3 (75.0)	13 (81.3)	0 (0.0)
Headache	1 (25.0)^a^	1 (25.0)	1 (25.0)	0 (0.0)	3 (18.8)	0 (0.0)
Presyncope	1 (25.0)^a^	0 (0.0)	0 (0.0)	0 (0.0)	1 (6.3)	0 (0.0)
Tongue paraesthesia	1 (25.0)	0 (0.0)	0 (0.0)	0 (0.0)	1 (6.3)	0 (0.0)
Psychiatric disorders	0 (0.0)	0 (0.0)	1 (25.0)	0 (0.0)	1 (6.3)	0 (0.0)
Low mood/anxiety	0 (0.0)	0 (0.0)	1 (25.0)	0 (0.0)	1 (6.3)	0 (0.0)
Renal and urinary disorders	0 (0.0)	0 (0.0)	0 (0.0)	0 (0.0)	0 (0.0)	1 (12.5)
Haematuria	0 (0.0)	0 (0.0)	0 (0.0)	0 (0.0)	0 (0.0)	1 (12.5)
Reproductive system and breast disorders	0 (0.0)	0 (0.0)	0 (0.0)	1 (25.0)	1 (6.3)	0 (0.0)
Dysmenorrhoea (menstrual symptoms)	0 (0.0)	0 (0.0)	0 (0.0)	1 (25.0)	1 (6.3)	0 (0.0)
Respiratory, thoracic and mediastinal disorders	1 (25.0)	1 (25.0)	0 (0.0)	0 (0.0)	2 (12.5)	3 (37.5)
Cough	0 (0.0)	0 (0.0)	0 (0.0)	0 (0.0)	0 (0.0)	1 (12.5)
Dyspnoea on exertion	0 (0.0)	1 (25.0)	0 (0.0)	0 (0.0)	1 (6.3)	0 (0.0)
Epistaxis	1 (25.0)	0 (0.0)	0 (0.0)	0 (0.0)	1 (6.3)	0 (0.0)
Hoarseness	0 (0.0)	0 (0.0)	0 (0.0)	0 (0.0)	0 (0.0)	1 (12.5)
Sinus disorder	0 (0.0)	0 (0.0)	0 (0.0)	0 (0.0)	0 (0.0)	1 (12.5)^a^
Skin and subcutaneous tissue disorders	0 (0.0)	1 (25.0)	0 (0.0)	0 (0.0)	1 (6.3)	0 (0.0)
Purpura	0 (0.0)	1 (25.0)	0 (0.0)	0 (0.0)	1 (6.3)	0 (0.0)
Vascular disorders	0 (0.0)	1 (25.0)	0 (0.0)	0 (0.0)	1 (6.3)	0 (0.0)
Cold hands	0 (0.0)	1 (25.0)	0 (0.0)	0 (0.0)	1 (6.3)	0 (0.0)
Unclassified	0 (0.0)	2 (50.0)	0 (0.0)	1 (25.0)	3 (18.8)	1 (12.5)
Brain fog	0 (0.0)	1 (25.0)	0 (0.0)	0 (0.0)	1 (6.3)	0 (0.0)
Dry mouth	0 (0.0)	0 (0.0)	0 (0.0)	1 (25.0)	1 (6.3)	0 (0.0)
Headache	0 (0.0)	0 (0.0)	0 (0.0)	0 (0.0)	0 (0.0)	1 (12.5)^a^
Loose stools	0 (0.0)	1 (25.0)	0 (0.0)	0 (0.0)	1 (6.3)	0 (0.0)

AE, adverse event; CTCAE, common terminology criteria for adverse events; IMP, investigational medicinal product; SOC, standard of care.

Percentages are based on the number of participants in the study arm. CTCAE v5.0 and associated SOC terms are used to classify AEs.

^a^Indicates a CTCAE version 5 grade 2 adverse event, all other adverse events are CTCAE version 5 grade 1.

### Analysis of secondary endpoints

There were no deaths, hospitalizations or any new requirement for oxygen therapy for any participant. Nineteen out of the 24 participants in the trial reported at least one AE, with 79% reporting a worst grade of 1 (*n* = 15) and 21% reporting a worst grade of 2 (*n* = 4). Fourteen of 16 (88%) on combination, and 5 of 8 (63%) standard-of-care participants had at least one AE. Combination therapy was generally well tolerated; Table 2 describes the frequencies of events across the groups. Although numbers were small, there did not appear to be any differences with the exception of dysgeusia (13/16; 81.3%) in participants receiving combination therapy compared with none in the standard-of-care arm. No SAEs were reported.

The confirmed PCR negativity rate at both Day 5 and Day 11 for combination therapy and standard of care was 68.8% (*n* = 11/16) and 62.5% (*n* = 5/8), respectively, with no significant differences observed at Days 2, 3 and 4. Changes in viral titres offer greater precision for estimating treatment effect. Posterior estimates from a bi-exponential model of virological clearance over time are shown in Figure 2c. The estimated mean of the treatment effect coefficient (γ_1_) is 1.57 (95% credible interval 1.22–2.07) for treatment effect. The posterior probability of the treatment effect coefficient being positive is >99.9%. This is above the pre-specified threshold of 91%, i.e. the treatment effect on the fast decay slope can be concluded. The sensitivity analysis of the model with the treatment effect on both fast and delay decay suggests a high confidence (87%) that the treatment effect coefficient for the fast decay is above 1.

### Pharmacokinetics

#### Plasma pharmacokinetics

For NHC, a total of 109 samples were obtained from 11 participants at Days 1 and 5 (10 evaluable, 1 participant with missing samples on Day 5 was excluded). Geometric mean (95% CI) AUC_0–4_ was 7054 (5581–8917) ng·h/mL on Day 1, and 7932 (6565–9585) ng·h/mL on Day 5. Geometric mean (95% CI) *C*_max_ was 3118 (2435–3992) ng/mL on Day 1, and 3335 (2764–4025) ng/mL on Day 5. *T*_max_ was 2.00 (range 1.00–4.00) h, and there was no evidence of any significant accumulation between Days 1 and 5.

For nirmatrelvir and ritonavir, 153 samples were obtained from 16 participants at Days 1 and 5. For nirmatrelvir, geometric mean (95% CI) AUC_0–4_ was 13 998 (10 671–18 361) ng·h/mL on Day 1, and 20 884 (17 624–24 747) ng·h/mL on Day 5. Geometric mean (95% CI) *C*_max_ was 5430 (4352–6775) ng/mL on Day 1, and 6414 (5412–7602) ng/mL on Day 5. *T*_max_ was 2.00 (range 2.00–4.00) h. For ritonavir, geometric mean (95% CI) AUC_0–4_ was 1076 (645–1975) ng·h/mL on Day 1, and 3063 (2327–4032) ng·h/mL on Day 5. Geometric mean (95% CI) *C*_max_ was 438 (295–651) ng/mL on Day 1, and 1129 (852–1498) ng/mL on Day 5. *T*_max_ was 2.00 (range 2.00–4.00) h. There was an approximately 50% increase in nirmatrelvir, and an approximately 3-fold increase in ritonavir exposure (*P* = 0.001 for both; Wilcoxon test) at Day 5 compared with Day 1

#### Non-plasma pharmacokinetics

**Figure 3.**
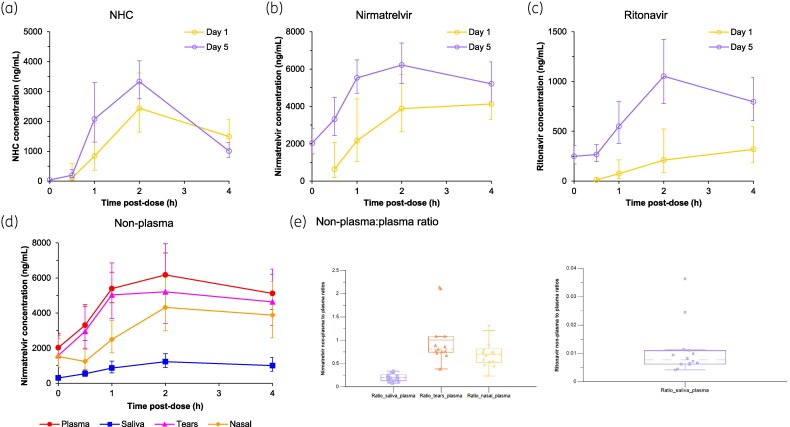
Geometric mean (95% CI) concentrations over nominal time post-dose of (a) NHC (*n* = 11), (b) nirmatrelvir (*n* = 16) and (c) ritonavir (*n* = 16) on study Day 1 (yellow) and Day 5 (purple) in plasma and in (d) non-plasma (plasma: circles; saliva: squares; tears: triangles; nasal secretions: diamonds) on Day 5 in COVID-19 patients administered prodrug molnupiravir (800 mg twice daily) and Paxlovid (nirmatrelvir/ritonavir 300/100 mg twice daily). (e) Boxplots of non-plasma to plasma ratios of nirmatrelvir (left) and ritonavir (right) are shown with data expressed as median and IQR (box), with mean (dashed line) and minimum and maximum values (whiskers); each point represents an individual ratio.

A total of 74 saliva, 73 tear and 74 nasal swabs were analysed from 15 participants (Figure 3). All saliva samples yielded measurable concentrations of nirmatrelvir, with geometric mean (95% CI) AUC_0–4_ and *C*_max_ of 3885 (2839–5317) ng·h/mL and 1294 (952–1758) ng/mL, respectively. Nirmatrelvir was detected in all tear samples, with geometric mean (95% CI) AUC_0–4_ and *C*_max_ of 17 558 (12 769–24 143) ng·h/mL and 6789 (4892–9421) ng/mL, respectively. Nirmatrelvir was also detected in all nasal secretions, with geometric mean (95% CI) AUC_0–4_ and *C*_max_ of 13 150 (9060–19 086) ng·h/mL and 4713 (3198–6946) ng/mL, respectively. For ritonavir, 1 of 15 pre-dose saliva samples at Day 5 was below the lower limit of quantitation. The geometric mean (95% CI) AUC_0–4_ and *C*_max_ were 28 (20–39) ng·h/mL and 10 (7–16) ng/mL, respectively. Given that ritonavir is not an active component of the regimen for efficacy, and that concentrations in saliva were low, we did not measure ritonavir in tears or nasal secretions. Compartmental penetration of nirmatrelvir calculated using plasma:compartment geometric mean AUC_0–4_ ratios (95% CI) was 0.18 (0.14–0.24) for saliva, 0.91 (0.69–1.19) for tears and 0.65 (0.50–0.83) for nasal secretions. The corresponding ratio for ritonavir was 0.009 (0.006–0.013).

## Discussion

Combination molnupiravir plus nirmatrelvir/ritonavir at licensed doses for 5 days is safe and well tolerated, with transient taste disturbance related to study drug. Despite a limited sample size, our model-based estimation of viral clearance was able to detect a significant difference in favour of the treatment arm over untreated participants. A larger study is required to compare virological efficacy and clinical effectiveness against monotherapy, recognizing the disconnect between these different outcome measures in clinical trials.

Importantly, plasma concentrations of NHC were within the range reported for our previous AGILE CST-2 trial.^26^ For nirmatrelvir, truncated pharmacokinetic profiles measured over 4 h at Day 5 (geometric mean AUC_0–4_ concentration 20 884 ng.h/mL) was similar to values provided in the drug label, with evidence of accumulation (plasma AUC_0–4_ ∼50% higher at Day 5 compared with Day 1, with a corresponding 3-fold rise in ritonavir exposure). It is unclear whether increased nirmatrelvir exposures resulted from accumulation of drug, or increasing boosting by ritonavir, or both. This contrasts with decreases of ritonavir over 14 days (as a consequence of autoinduction) when used to treat HIV. We have previously reported saliva, nasal and tear NHC concentrations of 3%, 21% and 22% that of plasma.^26^ Here we observed saliva, nasal and tear nirmatrelvir concentrations of 18%, 65% and 91% that of plasma, respectively (despite low penetration of ritonavir into saliva). These concentrations exceed the protein-adjusted EC_90_ for nirmatrelvir against SARS-CoV-2 of 292 ng/mL,^27^ suggesting good compartmental penetration of drug.

It is important to adequately characterize the safety of combination antivirals for SARS-CoV-2 infection, not only to allow testing in severe or persistent infections with currently circulating variants, but also to have ready-to-test regimens available for rapid evaluation in the event of any new variant, or new zoonotic transmissions where existing therapies become compromised. The approach taken here is pragmatic and cost-effective and could be extended to other antiviral combinations, including for emerging outbreaks beyond SARS-CoV-2.

## Supplementary Material

dkag180_Supplementary_Data

## Transparency declarations

S.H.K. has received research funding from ViiV Healthcare, Gilead Sciences, Pfizer and Merck Sharp & Dohme (MSD) for the Liverpool HIV Drug Interactions programme and for unrelated clinical studies. G.G. has received funding from Janssen-Cilag, AstraZeneca, Novartis, Astex, Roche, Heartflow, Celldex, BMS, BioNTech, MSD, IntraOp, Synairgen, Boehringer Ingelheim, Blood Cancer UK, Cancer Research UK, the NIHR, Asthma and Lung UK, UKRI, Wellcome Trust, NHS England, Unitaid, Imugene and GSK for unrelated academic clinical trials and programme funding, and has received honoraria/consulting fees from AstraZeneca and AbbVie. C.j.e. has received funding from AbbVie, Astra Zeneca, BMS, Gilead, Janssen Cilag, Lilly, Pfizer, MSD, Novartis, Sanofi and UCB. All other authors: none to declare. The AGILE CST-8 study was funded as acknowledged in the funding statement.

## Data sharing statement

The AGILE Trial Steering Committee will consider all reasonable requests by healthcare providers, investigators and researchers to provide anonymized data to address specific scientific or clinical objectives. The AGILE investigators are committed to reviewing requests from researchers for access to clinical trial protocols, de-identified patient-level clinical trial data, and study-level clinical trial data. Data will be assigned a DOI through deposition in the University of Liverpool Research Data Catalogue (rdm@liverpool.ac.uk) and shared under a Data Transfer agreement (or equivalent, e.g. as part of a research collaboration agreement or confidentiality disclosure agreement). Queries and requests for data access can be made by contacting livagile@liverpool.ac.uk.
